# Therapeutic Efficacy and Underlying Mechanisms of a Mannoglucan from *Hirsutella sinensis* Mycelium on Dextran Sulfate Sodium-Induced Inflammatory Bowel Disease in Mice: Modulation of the Intestinal Barrier, Oxidative Stress and Gut Microbiota

**DOI:** 10.3390/ijms252313100

**Published:** 2024-12-05

**Authors:** Weihua Ni, Yu Li, Jingyue Feng, Boxuan Liu, Hongyan Yuan, Guixiang Tai, Hongtao Bi

**Affiliations:** 1 Department of Immunology, College of Basic Medical Sciences, Jilin University, 126 Xinmin Street, Changchun 130021, Chinaliyu23@mails.jlu.edu.cn (Y.L.);; 2 Qinghai Provincial Key Laboratory of Tibetan Medicine Pharmacology and Safety Evaluation, Northwest Institute of Plateau Biology, CAS, 23 Xinning Road, Xining 810008, China

**Keywords:** *Hirsutella sinensis*, mannoglucan, colitis, oxidative stress, gut microbiota, intestinal immune, short-chain fatty acids

## Abstract

*Hirsutella sinensis (H. sinensis)*, a non-sexual form of the valuable Chinese medicinal herb, demonstrates various biological activities, such as immune modulation and antioxidative capabilities. Nonetheless, the effects of bioactive polysaccharides derived from *H. sinensis* on colitis have yet to be investigated. In our prior research, we extracted a mannoglucan (HSWP-1d) from *H. sinensis* and found that it attenuates TGF-β1-induced epithelial-mesenchymal transition. The present study investigated the protective effects of HSWP-1d against colitis induced by dextran sulfate sodium (DSS) in mice. The results demonstrate that HSWP-1d effectively ameliorates symptoms of colitis and preserves the intestinal barrier’s stability by enhancing the expression of tight junction proteins. The administration of HSWP-1d results in a reduction in oxidative stress through the augmentation of antioxidative enzyme activities, concomitant with the suppression of oxidative product generation. Simultaneously, HSWP-1d reduced the levels of pro-inflammatory cytokines while elevating the levels of anti-inflammatory cytokines, effectively mitigating the inflammatory response. Furthermore, HSWP-1d influences and alters short-chain-fatty-acid (SCFA) levels, thereby enhancing the intestinal microenvironment. In conclusion, HSWP-1d contributes to intestinal well-being and holds potential as both a therapeutic choice and a supplier of essential nutrients for the amelioration of colitis.

## 1. Introduction

Inflammatory bowel disease (IBD) is a kind of intestinal inflammatory disease which mainly includes ulcerative colitis and Crohn’s disease. IBD is often accompanied by a series of complications, such as perforation and bleeding, and in severe cases, it can lead to colon cancer and even threaten the patient’s life. The incidence of IBD is increasing globally. There are 10 million people living with IBD worldwide, and an estimated global prevalence of 5 million cases of ulcerative colitis was expected by 2023. Nevertheless, the current cure rate for IBD has not significantly improved [1,2].

Currently, the pathogenesis of IBD remains incompletely elucidated. However, it is widely acknowledged that IBD emerges as a consequence of the interplay of multiple mechanisms involving intestinal damage, oxidative stress, immune system dysfunctions, and alterations in the intestinal microbiota [3]. Under physiological conditions, tight junction (TJ) proteins and enteric epithelial cells work together to form a protective intestinal barrier that organizes the entry of deleterious agents, like intestinal bacteria and toxins, into the lamina propria of the gut mucosa, triggering an abnormal immune response [4]. However, during the development of IBD, TJ proteins decrease and the intestinal mucosal barrier function is impaired. This leads to the penetration of bacteria and their metabolites into the blood circulation through the intestinal mucosa, generating an abnormal immune response, which results in the release of significant amounts of inflammatory cytokines to defend against the infiltration of pathogens [5].

The overproduction of cytokines accompanied by the activation of immune cells, including Th17 cells and macrophages, may lead to the overproduction of reactive oxygen species [6,7]. In addition, oxidative stress disrupts the TJ proteins between epithelial cells [8], allowing more pathogens and toxins to invade the lamina propria of the intestinal mucosa and exacerbate the inflammatory response.

In addition, IBD causes a reduction in the population of ‘beneficial’ bacteria and a rise in the abundance of opportunistic pathogens in the body, disrupting the balance between host and commensal bacteria, affecting the level of metabolites and consequently the immune function in the intestinal microenvironment, leading to increased inflammation and sustained damage to intestinal tissues [9].

Repairing the gut barrier, mitigating the inflammatory response, and modulating the intestinal flora and its metabolites are essential for maintaining intestinal microenvironmental homeostasis. Current medications predominantly act by alleviating the inflammatory response [10], such as 5-aminosalicylic acid, corticosteroids, and immunosuppressive agents. Nevertheless, they prove ineffective and are frequently accompanied by considerable side effects, rendering them unsuitable for long-term usage [11]. Recently, considerable advancements have been made with novel therapeutic approaches such as biologics and fecal microbiota transplantation (FMT) [12]. However, these novel treatments are often costly and not effective for all patients, with potential side effects. Hence, there is an urgent requirement to develop highly effective and less toxic therapeutic strategies to improve IBD and enhance patient prognosis.

Plant- and fungus-based natural products are gaining more interest. They have various benefits and are less toxic. *Ophiocordyceps sinensis* is a renowned traditional Chinese health supplement and functional food, yet it is scarce and expensive. *Hirsutella sinensis* (*H. sinensis*), the asexual strain, exhibits similar biological activities and can, to some extent, serve as a substitute. The active components of *H. sinensis* also demonstrate various biological activities, such as enhancing immune cell function, modulating cytokine release [13], and exhibiting antioxidant properties [14]. Among these components, polysaccharides have been less studied. In a prior investigation, we isolated and extracted a novel mannoglucan, referred to as HSWP-1d, which was found to mitigate epithelial-mesenchymal transition (EMT) in vitro [15]. Inflammation is often linked to the initiation and modulation of EMT [16]. However, the anti-inflammatory properties of HSWP-1d have not been previously explored. Hence, in the present study, we explored the protective benefits of HSWP-1d against dextran sodium sulfate (DSS)-induced colitis in mice. This study explored the potential mechanisms by which HSWP-1d combats IBD through improving the intestinal mucosal barrier, modulating immune responses and oxidative stress, and regulating the gut microbiota. We hope that this research will provide new candidate supplment or health products derived from natural sources for the treatment of IBD.

## 2. Results

### 2.1. HSWP-1d Ameliorated Colitis Symptoms in Mice with DSS-Induced Colitis

**Figure 1:**
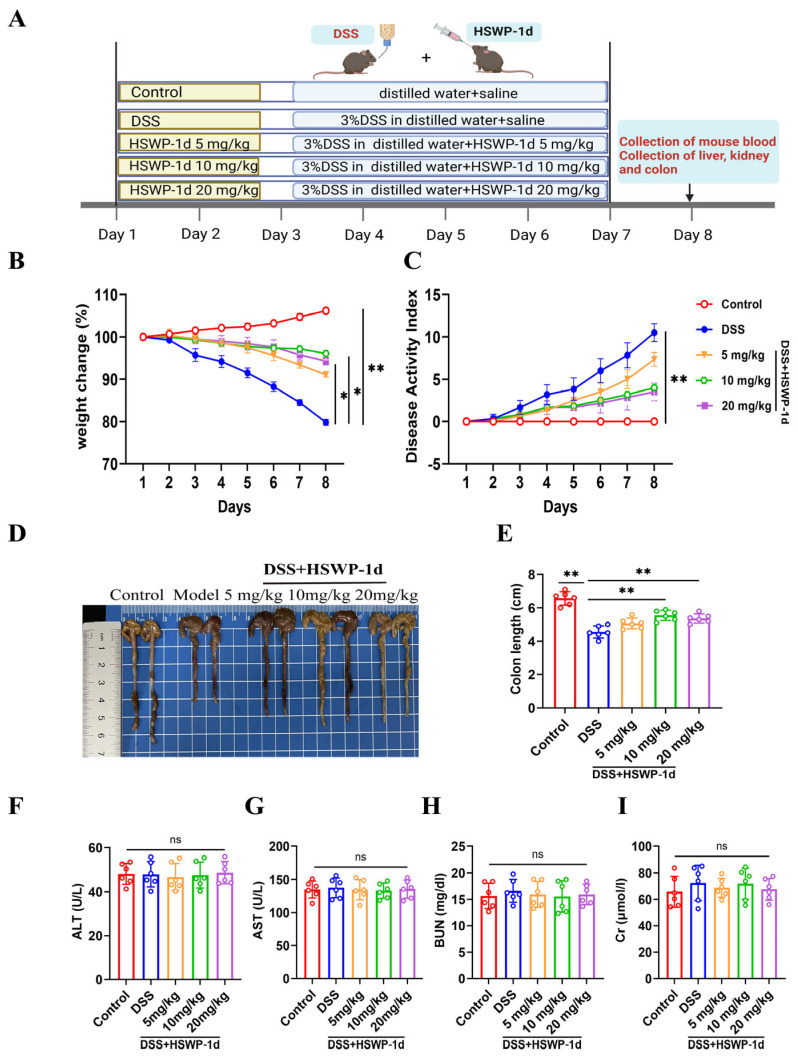
HSWP-1d ameliorated colitis symptoms in mice with DSS-induced colitis. (**A**) Experimental design diagram: mice were categorized into 5 groups (*n* = 6) receiving either plain water or water containing 3% DSS for a duration of 7 days while concurrently being administered HSWP-1d (5, 10, and 20 mg/kg) via intragastrical administration. (**B**) Changes of body weight. (**C**) Scores for the DAI. (**D**) Morphological assessment of the mouse colon. (**E**) Length measurements of the mouse colon. (**F**–**I**) Serum concentrations of ALT, AST, BUN, and Cr. Results are shown as means ± SDs, ns, not significant, *, *p* < 0.05; **, *p* < 0.01.

A DSS-induced colitis model was established in mice to evaluate the alleviating effect of intragastrical administration of HSWP-1d on IBD. During the entire experiment, mice that underwent DSS treatment displayed prominent pathological symptoms, such as weight loss, heightened severity of diarrhea, and rectal bleeding. In contrast, the administration of HSWP-1d at all doses (5, 10, and 20 mg/kg) resulted in improvements in these symptoms, with the medium and high doses demonstrating superior effects (Figure 1B,C). Additionally, colonic shortening was a common characteristic observed in DSS-induced colitis. Compared to findings for the DSS group, medium- and high-dose HSWP-1d significantly alleviated colonic shortening (*p* < 0.01). Low-dose HSWP-1d also showed an improvement in colonic shortening, although this was not statistically significant (Figure 1D,E). These findings indicate that HSWP-1d may have potential benefits in alleviating symptoms associated with colitis.

### 2.2. Preliminary Assessment of Hepatorenal Toxicity for HSWP-1d

Subsequently, we measured the levels of alanine aminotransferase (ALT)/aspartate aminotransferase (AST) and blood urea nitrogen (BUN)/creatinine (Cr) in the serum to assess liver and kidney damage for a preliminary toxicity evaluation.

The results showed no significant variations in the levels of ALT and AST (Figure 1F,G), as well as BUN and Cr (Figure 1H,I), among the groups. All values remained within the standard range, indicating that HSWP-1d, at the given dosage, did not cause liver and kidney toxicity.

### 2.3. HSWP-1d Improved Histopathological Damage and Intestinal Barrier Integrity

**Figure 2:**
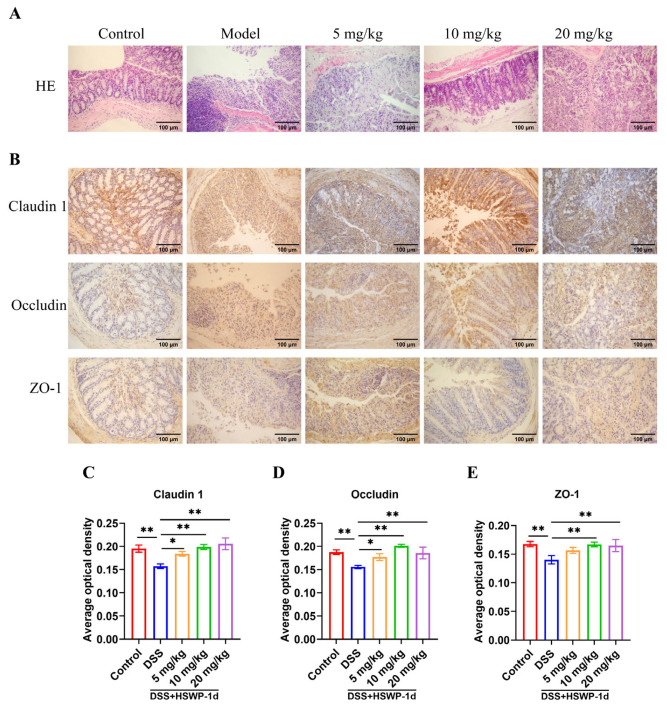
HSWP-1d ameliorated histopathologic damage and intestinal barrier damage in mice with DSS-induced colitis. (**A**) H&E staining of colon tissue. (**B**–**E**) Immunohistochemical assay and average integrated optical density of claudin 1, occludin, and ZO-1. Scale bar represents 100 µm. Results are shown as means ± SDs. *, *p* < 0.05; **, *p* < 0.01.

To further explore the impact of HSWP-1d on DSS-induced colitis, morphological variations in colon tissue by means of hematoxylin and eosin staining (H&E) were assessed. The results indicated that the DSS group displayed significant impairment to the intestinal mucosa, disrupted crypt architecture, and marked infiltration of inflammatory cells. In contrast, all three doses of HSWP-1d reduced mucosal injury and diminished inflammatory cell infiltration within the colon tissue (Figure 2A).

Claudin 1, occludin-1, and ZO-1, the major types of TJ proteins, play key roles in maintaining intestinal barrier function [17]. In mice with DSS-induced colitis, levels of TJ proteins were found to be decreased, leading to compromised epithelial integrity. However, administration of low, medium, and high doses of HSWP-1d significantly increased the expression levels of claudin 1 and occludin (*p* < 0.05). Additionally, medium and high doses of HSWP-1d also significantly elevated the level of ZO-1 (*p* < 0.01) (Figure 2B–E), thereby contributing to the enhancement of the intestinal barrier integrity. The findings suggest that HSWP-1d helped reduce intestinal tissue damage and restore the intestinal barrier, which may be closely related to maintaining intestinal homeostasis.

### 2.4. HSWP-1d Mitigated Oxidative Stress in DSS-Induced Colitis

**Figure 3:**
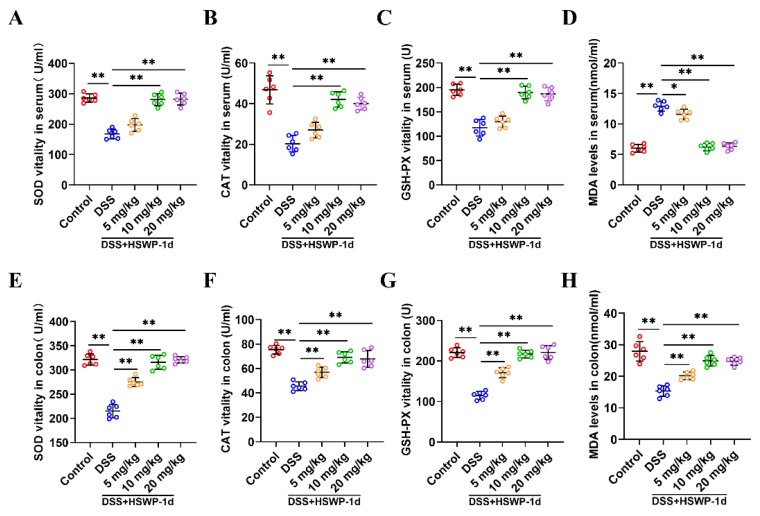
HSWP-1d mitigated oxidative stress in mice with DSS-induced colitis. (**A**–**H**) The activities of SOD, CAT, and GSH-Px, along with the MDA level, in both serum (**A**–**D**) and colon tissue (**E**–**H**) from colitis mice were measured. Results are shown as means ± SDs. *, *p* < 0.05; **, *p* < 0.01.

Oxidative stress is considered to be one of the pathophysiological mechanisms of IBD and a key factor in causing damage to intestinal tissue [18]. In this study, DSS treatment occasioned reduced the activity of antioxidant enzymes such as superoxide dismutase (SOD), catalase (CAT), and glutathione peroxidase (GSH-Px) and elevated the levels of the lipid peroxidation product malondialdehyde (MDA) in both serum and colon tissue. Nevertheless, medium and high doses of HSWP-1d significantly augmented the activities of these antioxidant enzymes and mitigated MDA levels (*p* < 0.01). Additionally, low doses of HSWP-1d also demonstrated comparable beneficial effects in the colon tissue (Figure 3A–H). The results imply that HSWP-1d strengthened the antioxidant defense system and reduced oxidation products, thereby minimizing oxidative damage in IBD.

### 2.5. HSWP-1d Reducd Pro-Inflammatory Cytokines While Enhancing IL-10 Levels in Mice with DSS-Induced Colitis

**Figure 4:**
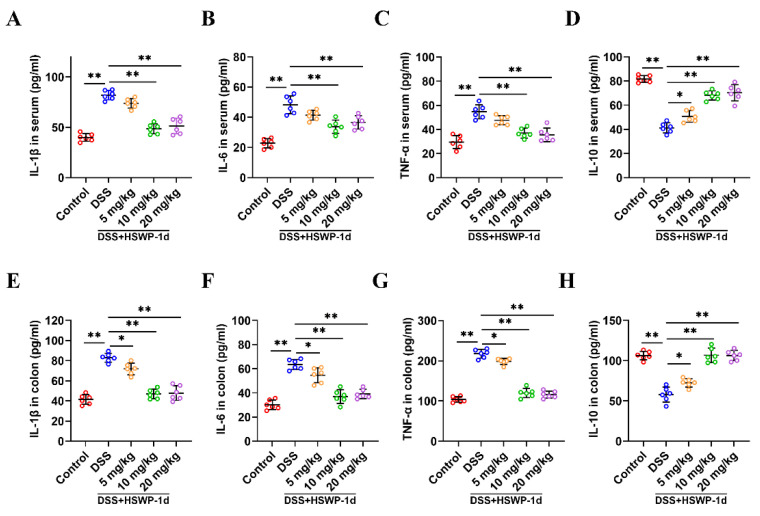
HSWP-1d regulated inflammatory cytokines in the serum and colon in mice with DSS-induced colitis. (**A**–**H**) The IL-1β, IL-6, TNF-α, and IL-10 concentrations in the serum (**A**–**D**) and colon tissue (**E**–**H**) of mice with colitis were measured. Results are shown as means ± SDs. *, *p* < 0.05; **, *p* < 0.01.

Inflammatory cytokines play a central role in the pathogenesis of IBD; therefore, we examined the levels of these cytokines [19]. Compared to findings for the control group, the administration of DSS led to elevated concentrations of pro-inflammatory cytokines (IL-1β, IL-6, TNF-α) and reduced levels of anti-inflammatory cytokine (IL-10) within both serum and colonic tissue. In contrast, the administration of HSWP-1d (10 and 20 mg/kg) effectively reversed the releasing effect of DSS on cytokines (*p* < 0.01) (Figure 4A–H). Notably, even low doses of HSWP-1d significantly adjusted the levels of cytokines affected by DSS-induced colitis in colon tissue (*p* < 0.05). These findings indicated that HSWP-1d had potential therapeutic effects on intestinal immune inflammation, which may contribute to the maintaining of gut immune homeostasis.

### 2.6. HSWP-1d Regulated the Composition of the Gut Microbiota and the Short-Chain-Fatty Acid (SCFA) Levels in Mice with DSS-Induced Colitis

#### 2.6.1. HSWP-1d Altered the Composition of the Gut Microbiota

**Figure 5:**
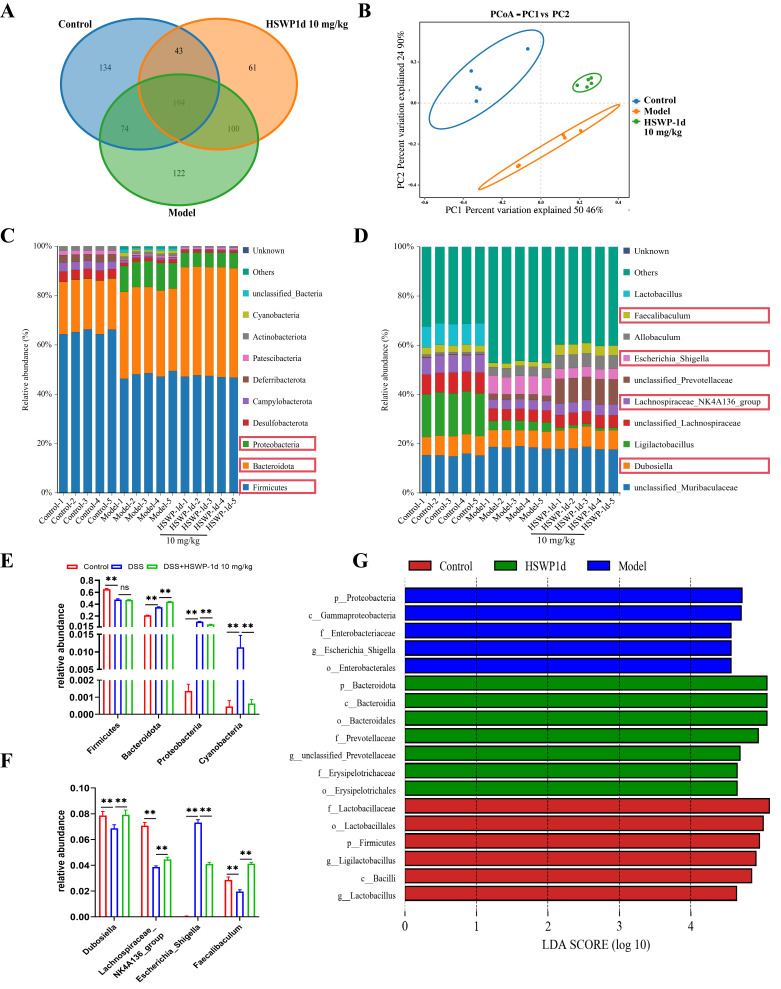
HSWP-1d influenced the intestinal microbiota composition in mice with DSS-induced colitis. (**A**) Venn diagram. (**B**) Principal-coordinate analysis (PCoA). (**C**,**D**) The distribution of gut microbiota at the phylum classification. (**E**,**F**) The breakdown of gut microbiota at the genus classification. (**G**) LDA scores for taxa with significant abundance differences (LDA > 4.5). Results are shown as means ± SDs, ns, not significant, **, *p*  < 0.01.

Dysbiosis of the gut microbiota is a key feature of IBD. DSS administration influenced 490 ASVs, and 398 ASVs responded to HSWP-1d treatment (Figure 5A). Principal-coordinate analysis (PCoA) was employed to evaluate alterations in the gut microbiota structure, and it indicated that the microbiota compositions in both the HSWP-1d and control groups were more comparable than those observed in the DSS group (Figure 5B). Subsequently, differential analyses were performed at the phylum and genus levels. A total of ten major bacterial phyla were identified, among which the contents of Firmicutes, Bacteroidetes, and Proteobacteria dominated, accounting for approximately 90% of the total. DSS administration significantly reduced the abundance of Firmicutes, while the levels of Bacteroidetes, Proteobacteria, and Cyanobacteria significantly increased (*p* < 0.01). HSWP-1d treatment significantly mitigated the increase in the abundances of Cyanobacteria and Proteobacteria induced by DSS (*p* < 0.01), but HSWP-1d did not influence the abundance changes of Firmicutes and Bacteroidetes caused by DSS (Figure 5C,E).

At the genus level, the DSS group manifested a notable reduction in the abundance of beneficial probiotics, including *Dubosiella*, *Faecalibaculum*, and *Lachnospiraceae_NK4A136_group*. In contrast, there was a notable increase in pathogenic bacteria linked to inflammation, such as *Escherichia_Shigella*. HSWP-1d notably elevated the abundance of these three probiotic species while reducing the levels of detrimental *Escherichia_Shigella* (*p* < 0.01) (Figure 5D,F), thereby safeguarding the intestinal barrier and mitigating inflammation.

With the aim of exploring of the differences in microbial community structural composition across various groups and to identify potential biomarkers, we performed LEfSe analysis. The predominant bacterial communities exhibited significant differences across the groups. An enrichment of pathogenic gut bacteria was observed in the DSS group, including Gammaproteobacteria, Enterobacteriaceae, and *Escherichia_Shigella*. Conversely, beneficial bacteria, including Bacteroidales, Prevotellaceae, and Erysipelotrichaceae, were more prevalent in the HSWP-1 group (Figure 5G).

These findings suggest that HSWP-1d aided in restoring the disrupted gut microbiota associated with IBD by promoting probiotics while suppressing harmful bacteria, thus helping to rebalance the microbiota.

#### 2.6.2. HSWP-1d Modulated SCFA Levels

**Figure 6:**
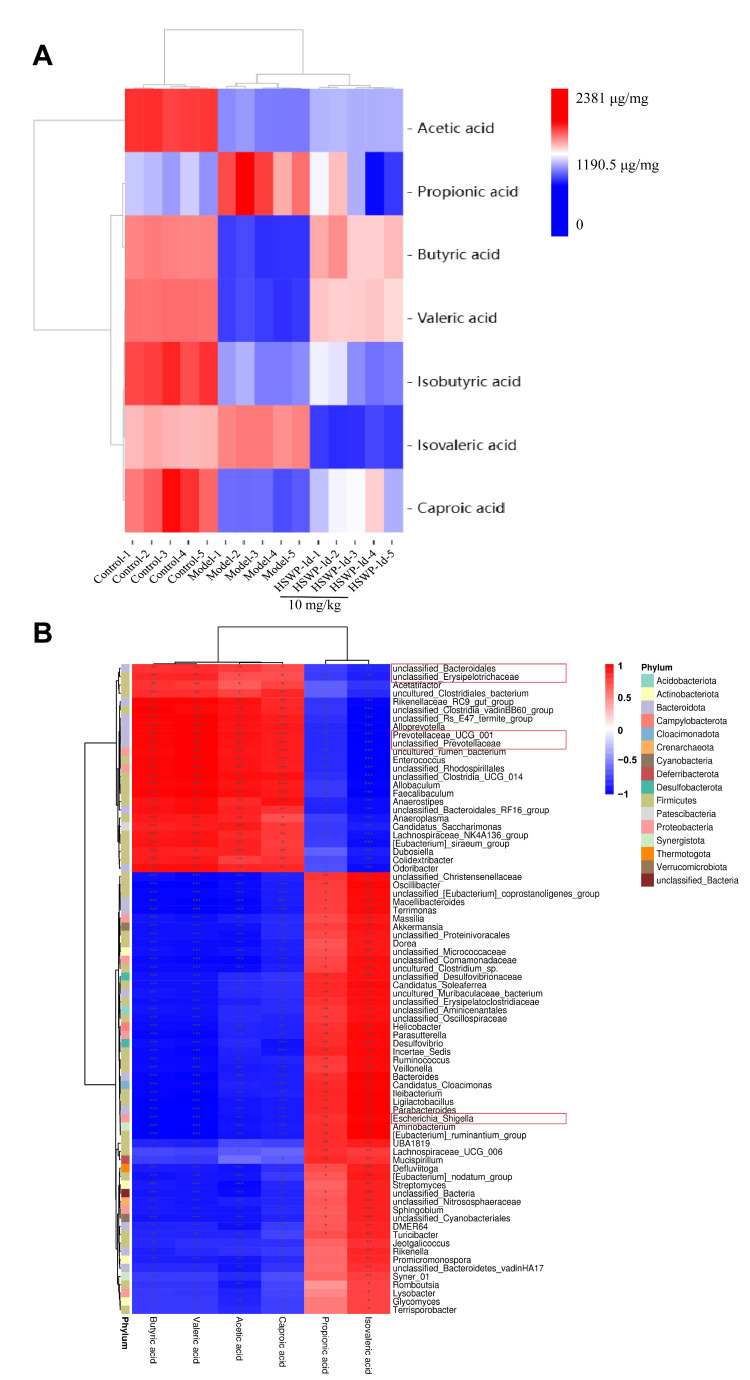
HSWP-1d influenced the structure and metabolites of intestinal flora in mice. (**A**) Heatmap of differentially abundant metabolite clustering among different samples (data were not normalized). (**B**) Correlation plot of the intestinal flora with metabolites between the DSS and HSWP-1d (10 mg/kg) groups. *, *p*  < 0.05; **, *p*  < 0.01; ***, *p*  < 0.001.

SCFAs, particularly acetic acid, propionic acid, and butyric acid, play a crucial role in maintaining intestinal homeostasis [20]. In this research, DSS notably decreased the concentrations of both acetic acid and butyric acid; conversely, HSWP-1d notably enhanced their levels (Figure 6A).

To examine the possible correlation between short-chain fatty acids and the gut microbiota, a heatmap was created to illustrate the correlation between SCFA concentrations and the abundance of gut microbes. The results showed that acetic acid, butyric acid, valerate, and hexanoate exhibited positive correlations with Bacteroidales, Prevotellaceae, and Erysipelotrichaceae while presenting negative correlations with *Escherichia_Shigella* (Figure 6B). These results indicate that HSWP-1d can restore SCFAs, like acetic acid and butyric acid, by altering the gut microbiota composition. This alteration may enhance gut health and resistance to inflammation.

## 3. Discussion

Compared to traditional treatments, polysaccharides have gained significant attention due to their remarkable efficacy in alleviating colitis and higher safety profiles [21]. Polysaccharides, especially those extracted from fungi, often possess unique structural characteristics and show great potential as functional food or health supplement ingredients [22]. As an asexual strain of *Ophiocordyceps sinensis*, *H. sinensis* is a medicinal and edible fungus that serves as a cost-effective alternative to its active components and is often used to treat organ transplant rejection [23]. In our previous study, we isolated a bioactive glucomannan, HSWP-1d, from *H. sinensis*, which has been shown to alleviate EMT. These activities are usually related to anti-inflammatory activity. Therefore, in this study, we explored its potential in treating colitis.

DSS-induced colitis is a well-recognized animal model, although it may not fully represent the entire pathogenic process of human IBD [24]. However, it mimics the biological consequences resulting from intestinal barrier damage, innate immune abnormalities, and dysbiosis that are highly relevant to human IBD [24,25]. In the present research, a mouse colitis model was induced using DSS to evaluate the therapeutic and alleviative efficacy of HSWP-1d on IBD and to explore its underlying mechanisms. The results showed that HSWP-1d had a protective effect on mice with IBD: it significantly alleviated the pathological damage and symptoms of the intestine in mice with IBD. Other literature indicates that konjac glucomannan also exhibits anti-inflammatory effects on IBD [26]. These findings indicate that HSWP-1d may have potential benefits in alleviating symptoms associated with colitis.

The emergence and cure of IBD are consequences of the interplay of multiple mechanisms, including regulation of intestinal barrier, oxidative stress, and alterations in the intestinal microbiota, and these mechanisms also interact with one another. Oxidative stress perturbs cellular homeostasis, giving rise to mucosal barrier dysfunction, immune response imbalance, and gut microbiota dysbiosis [8,27]. Immune cytokines act as a barrier of gut immunity, and their aberrant expression has the potential to disrupt the TJ proteins between epithelial cells and modify gut permeability, thereby aggravating IBD symptoms via pathological cascades [28]. Our results indicate that HSWP-1 restores the reduced expression of TJ proteins caused by DSS, consistent with other studies demonstrating that glucomannan from aloe vera gel maintains the integrity of the intestinal barrier [29]. The findings further substantiate that HSWP-1d aids in restoring intestinal barrier function, thereby alleviating symptoms of colitis.

Additionally, HSWP-1d mitigates oxidative stress induced by DSS. It also decreases the secretion of pro-inflammatory factors while upregulating the anti-inflammatory factor IL-10. Glucomannan from konjac has demonstrated effects in reducing oxidative stress and modulating inflammatory responses [30]. These results indicate that the mechanism of HSWP-1d for the alleviation of IBD involves oxidative stress and the regulation of inflammatory factors to maintain and restore intestinal homeostasis.

Dysbiosis of the gut microbiota is also one of the main factors in the occurrence and development of colitis. Imbalance in the gut microbiota can lead to impaired intestinal barrier function and increased mucosal permeability, resulting in invasion by harmful substances and triggering excessive inflammation, which undermines the body’s immune tolerance to gut microbes and exacerbates colitis [31]. In our study, DSS significantly increased the abundance of harmful bacteria such as Proteobacteria, Cyanobacteria, and *Escherichia_Shigella*. The rise in Proteobacteria is associated with oxidative stress and colitis severity [32]. *Escherichia_Shigella* disrupts intestinal barrier function by degrading mucin, while Cyanobacteria’s pro-inflammatory activity is linked to IBD progression [33,34]. However, after treatment with HSWP-1d, the abundance of these three harmful bacterial groups significantly decreased. Meanwhile, HSWP-1d also increased the abundance of beneficial bacteria such as *Dubosiella*, *Faecalibaculum*, and *Lachnospiraceae_NK4A136_group*. *Dubosiella* is a gut probiotic with anti-inflammatory benefits for IBD [35]. *Faecalibaculum* is a biomarker linked to oxidative stress, beneficial for colitis [36], while *Lachnospiraceae_NK4A136_group* aids in alleviating colitis-related inflammation and oxidative stress [37]. All three beneficial bacteria produce butyrate, supporting epithelial cell renewal and maintaining intestinal barrier integrity [35,38,39].

Beyond direct mechanisms, the intestinal microbiota can also influence health by fermenting complex polysaccharides to generate metabolites. HSWP-1d is a mannoglucan that predominantly occurs as soluble, viscous fibers [40]. Due to the typical inability of human enzymes (including amylase) to hydrolyze this type of carbohydrate, mannoglucans are generally transported to the colon, where they undergo fermentation by a diverse array of gut bacteria [41]. This fermentation process produces metabolites such as SCFAs. SCFAs are essential for improving the integrity and maintaining intestinal homeostasis. On one hand, they directly serve as an indispensable energy source for intestinal epithelial cells [42] and enhance the expression of tight junction (TJ) proteins [43]. Additionally, SCFAs may correlate with a decreased pH in the intestinal lumen, thereby suppressing the colonization of harmful microorganisms [44]. Our findings suggest that HSWP-1d elevates levels of SCFAs, particularly butyric acid and acetic acid, and increases the expression of TJ proteins, indicating that HSWP-1d may directly impact the progression of IBD through SCFAs. On the other hand, SCFA modulation can indirectly influence the progression of IBD through interactions with antioxidant and anti-inflammatory effects [45]. HSWP-1d has been shown to elevate levels of butyric acid and acetic acid. Butyric acid has been demonstrated to enhance antioxidant enzymes such as GSH-Px, which are vital for reducing oxidative stress [46,47]. Both butyric acid and acetic acid exhibit anti-inflammatory effects and may alleviate inflammation during the progression of IBD [46,48]. Furthermore, HSWP-1d has shown the ability to modulate oxidative stress and alleviate inflammatory responses. Therefore, the regulation of SCFA production by HSWP-1d may contribute to its anti-inflammatory and antioxidant characteristics.

The functionality of polysaccharides is affected by factors such as their molecular weight, the types of monosaccharide units, the characteristics of glycosidic bonds, the level of branching, and their three-dimensional spatial arrangement. HSWP-1d is primarily composed of mannoglucan, which consists mainly of mannose and glucose. In addition to fungi, mannoglucan can be isolated from konjac and yeast [30,49]. Due to its unique glycosidic bonds, highly branched structure, and specific composition and ratios of monosaccharides, HSWP-1d not only exerts its effects by being degraded into SCFAs but also may have the potential to directly bind to the surface TLR receptors of immune cells, such as macrophages and regulatory T cells (Tregs) [50,51]. This interaction is also closely linked to its diverse biological activities, including immunomodulation, antioxidant effects, and prebiotic functions [52]. We will discuss this research further in our follow-up study.

Furthermore, the biological activity of biopharmaceuticals usually depends on several factors, including the dosage, administration route, excipients, and timing, all of which can influence their effects. In our study, we employed a dosage range of 5–20 mg/kg, which is relatively low for polysaccharide treatments in IBD [53]. Notably, a dosage of 5 mg/kg already demonstrated effects, which is significant for the extraction of effective components, cost of use, and control of toxicity. Moving forward, we will conduct a thorough investigation into the toxicity associated with higher doses and further explore the comparative and synergistic effects of other IBD therapeutic strategies, such as 5-aminosalicylic acid, in their role as immunomodulators or adjunctive therapies. Additionally, we will perform acute and chronic toxicity analyses to provide a theoretical basis for clinical applications.

## 4. Materials and Methods

### 4.1. Reagent and Materials

Polysaccharide fraction HSWP-1d was kindly provided by Qinghai Provincial Key Laboratory of Tibetan Medicine Pharmacology and Safety Evaluation, Northwest Institute of Plateau Biology, CAS. It was isolated from the mycelia of *H. sinensis* (with the strain identification number 2011-WEIJIANZI-No.132 from the Institute of Microbiology, CAS) and was identified as a mannoglucan (Mw 1.12 kDa), as previously described in our research [15].

DSS was provided by Shanghai Aladdin Biochemical Technology Co., Ltd., in Shanghai, China. High-definition H&E staining kits, diaminobenzidine (DAB) chromogenic reagents, and CAT test kits were provided by Wuhan Servicebio Technology Co., Ltd., based in Wuhan, Hubei Province, China. Nanjing Jiancheng Biotechnology Research Institute Co., Ltd., in Nanjing, Jiangsu Province, China, supplied the assay kits for GSH-Px, MDA, and SOD. ALT, AST, BUN, and Cr test kits were provided by Changchun Huili Biotech Co., Ltd., in Changchun, Jilin Province, China. ELISA kits for TNF-α, IL-1β, IL-6, and IL-10 were provided by R&D Systems, headquartered in Minneapolis, MN, USA. Antibodies specific to ZO-1, occludin, and claudin 1 were provided by Proteintech Group, Inc., located in Wuhan, Hubei Province, China. Tiangen Biotech Co., Ltd., in Beijing, China, provided the TGuide S96 magnetic stool DNA extraction kit, while the Omega DNA purification kit was purchased from Omega, Inc., in Atlanta, GA, USA.

### 4.2. Experimental Design

Thirty male C57BL/6J mice (6–8 weeks old, weighing 20 ± 2 g) were procured from Yisi Laboratory Animal Technology Co., Ltd., Changchun, China. The animal experimental protocols were approved by Jilin University (permit number: 2023322). Following a 7-day acclimation period under standard conditions (temperature of 22 ± 2 °C, relative humidity of 55–60%, and a 12-h light/dark cycle), the mice were randomly divided into 5 groups (*n* = 6): control, DSS, DSS + HSWP-1d at a low dose of 5 mg/kg, DSS + HSWP-1d at a medium dose of 10 mg/kg, and DSS + HSWP-1d at a high dose of 20 mg/kg. To induce colitis, all groups except the control group received oral administration of 3% DSS for 7 days. Subsequently, HSWP-1d was dissolved in saline. The groups treated with HSWP-1d underwent daily intragastrical administration of HSWP-1d (5, 10, and 20 mg/kg) for 7 days; meanwhile, the control and DSS groups received an equal volume of saline. A schematic overview of the animal experiments is presented in Figure 1A.

### 4.3. Sample Collection

Throughout the experiment, daily observations were conducted on the mice to track variations in body weight, stool texture, and any blood present in their feces for the purpose of determining the DAI [53]. Fresh stool samples were collected on day 7. On day 8, mice were anesthetized with ether and blood samples were collected and centrifuged at 4 °C at a speed of 3000× *g* for 20 min to obtain serum. Mice were euthanized and colon, liver, and kidney samples were harvested. Fresh feces, serum, and proximal colon tissues were preserved at −80 °C, while the distal colon, liver, and kidneys were subjected to fixation in 4% paraformaldehyde.

### 4.4. H&E and Immunohistochemical Staining (IHC)

H&E and IHC staining were conducted on distal colonic segments. The tissues were fixed in 4% paraformaldehyde, embedded in paraffin, sliced into sections, deparaffinized, and then stained with H&E. Histological alterations were examined using an optical microscope (OLYMPUS BX51, Olympus Corporation, Tokyo, Japan).

Paraffin sections were deparaffinized, rehydrated, and subjected to antigen retrieval and blocking procedures. Primary antibodies targeting ZO-1, occludin, and claudin 1 were applied and incubated overnight at 4 °C. Following this, the sections were incubated with HRP-conjugated secondary antibodies at room temperature for 50 min, after which DAB staining and hematoxylin counterstaining were performed. Observations were conducted using an optical microscope (OLYMPUS BX51, Olympus Corporation, Tokyo, Japan), and data were analyzed with ImageJ software (version 15.4f).

### 4.5. Cytokines

Colon tissues were processed in PBS to create a homogenate and then centrifuged at 5000× *g* for 10 min to collect the supernatant. Subsequently, ELISA kits were employed to analyze the levels of IL-1β, IL-6, TNF-α, and IL-10 measured in serum as well as in homogenates of colon tissue.

### 4.6. Biochemical Indicators

Biochemical assay kits were utilized to assess the activity levels of CAT, SOD, and GSH-Px as well as MDA in serum and colon tissue. In addition, serum levels of ALT, AST, BUN, and Cr were measured using a biochemical analyzer (Rayto Life and Analytical Sciences Co., Ltd., Shenzhen, China) to assess the toxicity of HSWP-1d to the liver and kidney.

### 4.7. S rDNA Sequencing of the Gut Microbiota

Genomic DNA from fecal samples was extracted utilizing the TGuide S96 magnetic fecal genomic DNA kit. The V3–V4 segment of bacterial 16S rRNA was amplified through polymerase chain reaction (PCR) using primers 338F (5′-ACTCCTACGGGAGGCAGCA-3′) and 806R (5′-GGACTACHVGGGTWTCTAAT-3′). Following amplification, the PCR products were purified with the Omega DNA purification kit and quantified. The samples were normalized to prepare sequencing libraries, after which high-throughput sequencing was conducted using the Illumina NovaSeq 6000 platform (Illumina, San Diego, CA, USA). Data denoising was performed using the dada2 method within QIIME2 version 2020.6 software. ASVs were established, followed by analyses of diversity, differential expression, and correlation assessments.

### 4.8. Profiling of SCFAs

Place the collected fecal sample in a 1.5-mL centrifuge tube and add 500 μL of water along with 100 mg of glass beads. Homogenize the mixture for 1 minute, and then centrifuge at 12,000 rpm for 10 min at 4 °C. Afterward, collect 200 μL of the supernatant and mix it with 100 μL of a 15% phosphoric acid solution, followed by adding 20 μL of an internal standard (4-methylvaleric acid) at a concentration of 375 μg/mL and then adding ether (280 μL). Homogenize again for one minute before performing another round of centrifugation under the same conditions for ten minutes. Finally, collect the supernatant to measure SCFA concentrations in feces using gas chromatography.

### 4.9. Statistical Analysis

Statistical analysis was carried out using GraphPad Prism 10.0 software, utilizing one-way analysis of variance (ANOVA). The results are presented as means ± standard deviations (SD), and all experiments were replicated in triplicate. A *p* value of <0.05 was deemed statistically significant.

## 5. Conclusions

The novel mannoglucan HSWP-1d derived from *H. sinensis* has demonstrated significant anti-IBD effects by influencing various systemic pathways. Our research uncovered its mechanisms, which include the modulation of oxidative stress and inflammatory mediators, as well as the regulation of gut microbiota composition and SCFA metabolites. These results indicate that HSWP-1d presents promising therapeutic potential for IBD treatment. Nonetheless, further research is needed to fully comprehend the complex interactions and pathways underlying these effects. This will help establish a basis for its potential use as a health supplement or functional food in treating IBD, ultimately leading to the development of more precise and efficient therapies for individuals with this condition.

## Data Availability

Data are contained within the article.

## Abbreviations

IBD	inflammatory bowel disease
TJ	tight junction
*H. sinensis*	*Hi* *rsutella sinensis*
DSS	Dextran sulfate sodium
ALT	alanine aminotransferase
AST	aspartate aminotransferase
SOD	superoxide dismutase
CAT	catalase
GSH-Px	glutathione peroxidase
MDA	malondialdehyde
SCFA	short-chain fatty acid
